# Traditional Chinese medicine nursing competency and its correlates among clinical nurses in Guangyuan City, China

**DOI:** 10.3389/fmed.2026.1820919

**Published:** 2026-04-16

**Authors:** Ming Jin, Jing Yang, Yiman Hou, Li Zhang, Juan Zheng, Yongping Lu, Zhiqiang Wang

**Affiliations:** 1 Guangyuan Central Hospital, Guangyuan, Sichuan, China; 2 Guangyuan Hospital of Traditional Chinese Medicine, Guangyuan, Sichuan, China; 3 School of Medicine, Yangtze University, Jingzhou, Hubei, China

**Keywords:** clinical nurses, cross-sectional survey, nursing competency, traditional Chinese medicine nursing, training needs

## Abstract

**Objective:**

To assess Traditional Chinese Medicine (TCM) nursing competency among clinical nurses in Guangyuan City, China, and identify associated factors to inform targeted training and management.

**Methods:**

A cross-sectional survey was conducted from January to October 2024. Using multistage stratified cluster sampling, clinical nurses were recruited from 20 public medical institutions in Guangyuan City. Data were collected using a general information questionnaire, the TCM Nursing Competency Scale, and a TCM nursing training needs questionnaire. Analyses were performed in SPSS 25.0. Variables significant in univariate analyses were entered into a multiple linear regression model (two-tailed *α* = 0.05).

**Results:**

Of 1,300 questionnaires distributed, 1,210 were valid (93.08%). The mean overall competency score was 3.19 ± 0.91 (moderate). Dimension scores were highest for TCM nursing techniques (3.43 ± 0.97) and lower for foundational theory (3.07 ± 0.92) and advanced clinical practice (3.07 ± 1.01). Regression analysis showed that hospital type, perceived training volume/adequacy, educational level, managerial emphasis on TCM nursing training, number of TCM techniques nurses could perform, and willingness to participate in TCM nursing training were associated with overall competency (all *p* < 0.05). The final model was statistically significant (*R*^2^ = 0.473, adjusted *R*^2^ = 0.470; *F* = 153.931, *p* < 0.001).

**Conclusion:**

Nurses in Guangyuan City showed moderate and uneven TCM nursing competency. Both individual characteristics and organizational training conditions were associated with competency, suggesting the potential value of targeted training and strengthened institutional support.

## Introduction

Traditional Chinese Medicine (TCM) nursing is an integral component of the TCM service system, emphasizing holistic care and syndrome differentiation to guide nursing assessment, interventions, and health education through appropriate TCM techniques (1).

With the expansion of TCM nursing services and the development of specialty nursing, higher levels of TCM nursing competency are increasingly required in clinical practice (2). Insufficient competency may compromise the standardized delivery of TCM nursing techniques and limit their clinical effectiveness.

TCM nursing competency is generally conceptualized as a multidimensional construct encompassing foundational TCM theory, proficiency in commonly used TCM nursing techniques, and advanced clinical practice capabilities (3–5). Existing evidence suggests that competency development is influenced by both individual characteristics (e.g., educational level, training engagement, and prior practice experience) and organizational factors, including institutional type and level, availability of training opportunities and practice platforms, and the degree of managerial support for TCM nursing training (6). Limited organizational support may restrict training and practice exposure, whereas stronger support may facilitate sustained competency improvement.

However, evidence regarding factors associated with TCM nursing competency remains inconsistent across regions and institutional tiers. Prior studies have largely focused on developed eastern regions or tertiary hospitals, with relatively limited systematic assessments conducted in less developed areas and across multi-tiered healthcare institutions (7). This gap may constrain the generalizability of current findings.

Guangyuan City in northern Sichuan is characterized by a mixed healthcare system that includes both general and TCM hospitals across different institutional levels. Under relatively constrained healthcare resources, the status and determinants of nurses’ TCM nursing competency in this region remain insufficiently described (8–12). Therefore, this cross-sectional study used multistage stratified cluster sampling to investigate TCM nursing competency and training needs among clinical nurses from 20 public medical institutions in Guangyuan City. Multiple linear regression was performed to examine factors associated with TCM nursing competency, aiming to inform targeted training strategies and nursing human resource management.

## Materials and methods

### Study design and participants

This cross-sectional survey was conducted from January to October 2024. Clinical nurses were recruited from 20 public medical institutions in Guangyuan City using a multistage stratified cluster sampling approach (13–17).

Inclusion criteria were: (1) holding a valid nursing license; (2) having at least 1 year of clinical nursing experience; and (3) being currently involved in TCM nursing techniques or related services.

Exclusion criteria were: (1) continuous absence for ≥3 months (e.g., sick leave, personal leave, or maternity leave) during the survey period; and (2) trainees or interns who were not formally registered with the institution. In addition, questionnaires completed in <3 min, showing evident response patterns, or failing logical consistency checks were excluded from analysis.

### Survey instruments

#### General information questionnaire

A general information questionnaire was developed by the research team based on a literature review and expert consultation. It collected participants’ demographic and professional characteristics, including sex, age, years of service, professional title, employment status, highest educational level, graduating institution, department, and the types/number of TCM nursing techniques the nurse was able to perform (18–21).

#### TCM nursing competency scale

TCM nursing competency was assessed using the TCM Nursing Competency Scale developed by Wang et al. (22). The scale includes 37 items across three dimensions: fundamental TCM nursing knowledge (12 items), TCM nursing techniques (18 items), and advanced clinical TCM nursing practice (7 items). Items are rated on a 5-point Likert scale from 1 (“very unfamiliar”) to 5 (“very familiar”), with higher scores indicating higher competency. The original scale demonstrated good internal consistency (Cronbach’s *α* = 0.91, 0.88, and 0.96 for the three dimensions) and acceptable content validity (CVI = 0.80–0.91). In the current study, the internal consistency of the scale was re-evaluated in the study sample using Cronbach’s *α* coefficients. The Cronbach’s *α* coefficients were 0.944 for the fundamental TCM nursing knowledge dimension, 0.913 for the TCM nursing techniques dimension, and 0.912 for the advanced clinical TCM nursing practice dimension, indicating excellent internal consistency in the present sample.

#### TCM nursing training needs survey questionnaire

Training needs were evaluated using the “Survey Questionnaire on Training Needs for TCM Nursing Personnel in Public Hospitals” developed by Ye et al. (23). The questionnaire contains six items covering willingness to participate in training, perceived importance of training, desired training volume, preferred theoretical training methods, preferred skills training methods, and desired training content. In the pilot assessment, the questionnaire showed good reliability (Cronbach’s *α* = 0.84) and acceptable construct validity, with a cumulative variance explained of 62.1% (24–27).

#### Sample size and sampling method

Sample size was estimated based on Kendall’s rule of thumb, recommending a sample size of 5–10 participants per item (28, 29). Given a total of 53 items across the study questionnaires and assuming a 20% non-response rate, the minimum required sample size was 318.

A stratified cluster sampling method was applied. First, public medical institutions in Guangyuan City were stratified by hospital grade and type (general hospitals vs. TCM hospitals). Second, 20 hospitals were selected proportionally, including seven tertiary and 13 secondary hospitals. Third, eligible clinical nurses within the selected hospitals were sampled by clusters to complete the survey (9, 30). Overall, 1,300 questionnaires were distributed and all were returned. After quality control screening, 90 questionnaires were excluded, including 38 completed in less than 3 min, 30 with evident straight-line or patterned responses, 14 that failed logical consistency checks, and 8 with substantial missing data or duplicate submissions. Finally, 1,210 valid questionnaires were included in the analysis, yielding a valid response rate of 93.08%.

#### Data collection and quality control

Data were collected using anonymous electronic questionnaires administered by uniformly trained investigators from January to October 2024. Before data collection, the study objectives and procedures were explained to hospital administrators, and the survey was implemented after institutional consent was obtained. Participation was voluntary, and informed consent was obtained from all participants. No personally identifiable information was collected. To enhance data quality, each IP address was allowed a single submission, and questionnaires were screened using completion time, response pattern checks, logical consistency rules, and data completeness. A total of 1,300 questionnaires were distributed and all were returned. Of these, 90 questionnaires were excluded, including 38 completed in less than 3 min, 30 with evident straight-line or patterned responses, 14 that failed logical consistency checks, and 8 with substantial missing data or duplicate submissions. Finally, 1,210 valid questionnaires were retained for analysis, yielding a valid response rate of 93.08%. After screening, data were exported in a standardized format for statistical analysis.

#### Statistical analysis

All analyses were performed using SPSS 25.0. Normality of continuous variables was assessed using the Kolmogorov–Smirnov test (31). Normally distributed variables are presented as mean ± standard deviation, and non-normally distributed variables as median (interquartile range). Between-group comparisons were conducted using independent-samples *t* tests or one-way analysis of variance (ANOVA) for normally distributed data with homogeneity of variance, and nonparametric rank-sum tests for non-normally distributed data. Categorical variables are presented as frequency (percentage).

The overall TCM nursing competency score was treated as the dependent variable. The internal consistency reliability of the TCM Nursing Competency Scale in the current sample was assessed using Cronbach’s *α* coefficients. Variables with statistical significance in univariate analyses were entered into a multivariable linear regression model using the enter method to identify factors associated with competency. Model fit was evaluated using *R*^2^, adjusted *R*^2^, and the *F* statistic. Multicollinearity was assessed using tolerance and variance inflation factor (VIF), and independence of residuals was evaluated using the Durbin–Watson statistic. Residual diagnostics were further examined using histograms, normal P–P plots, and scatterplots of standardized residuals against standardized predicted values to assess the assumptions of residual normality, linearity, and homoscedasticity. A two-tailed *p* value <0.05 was considered statistically significant.

#### Ethical principles

This study involved no intervention and followed the ethical principles of respect for persons, beneficence, and justice as outlined in the Belmont Report (32–36). All participants provided informed consent and were free to withdraw at any time. The survey was completed anonymously, and all data were used solely for research purposes and kept confidential. Ethical approval was obtained from the Ethics Committee of Guangyuan Central Hospital (Approval No. GYZXLL2024010).

## Results

### Overall level of traditional Chinese medicine nursing competence

A total of 1,210 valid questionnaires were included. The TCM Nursing Competency Scale demonstrated excellent internal consistency in the current sample, with Cronbach’s *α* coefficients of 0.944 for the fundamental TCM nursing knowledge dimension, 0.913 for the TCM nursing techniques dimension, and 0.912 for the advanced clinical TCM nursing practice dimension. The mean total score for TCM nursing competency among participants was (3.19 ± 0.91), indicating a moderate level. Scores across dimensions, from highest to lowest, were: Common TCM Nursing Techniques (3.43 ± 0.97), Fundamental TCM Theoretical Knowledge (3.07 ± 0.92), and Advanced TCM Clinical Practice (3.07 ± 1.01) (see Table 1). Overall, the structure exhibited relatively higher technical competence alongside relatively weaker theoretical and advanced practical capabilities.

**Table 1:** TCM nursing competency scores (*n* = 1,210).

Item	Score	95% confidence interval for mean
Basic TCM theoretical knowledge	3.07 ± 0.92	3.07 (3.02, 3.12)
Common traditional Chinese nursing techniques	3.43 ± 0.97	3.43 (3.37, 3.48)
Advanced TCM nursing clinical practice	3.07 ± 1.01	3.07 (3.02, 3.13)
Total traditional Chinese medicine nursing competency score	3.19 ± 0.91	3.19 (3.14, 3.24)

### Item-level performance in fundamental TCM theoretical knowledge

Item scores within the Fundamental TCM Theoretical Knowledge dimension varied (Table 2). The highest-rated items were Four Diagnostic Methods (3.32 ± 1.08) and Yin–Yang and Five Elements (3.16 ± 0.97), whereas the lowest-rated items were Interpretation of Traditional Chinese Medical Classics (2.73 ± 1.06) and Basic Knowledge of Prescriptions/Formulas and Medicinal Substances (2.82 ± 1.05).

**Table 2:** Scores for basic TCM theoretical knowledge competency (*n* = 1,210).

Item	Overall	95% confidence interval
Yin-Yang and five elements	3.16 ± 0.97	3.16 (3.11, 3.22)
The Theory of Zang-Xiang	2.91 ± 1.05	2.92 (2.86, 2.97)
Essence, Qi, Blood, and Body Fluids	3.00 ± 1.01	3.00 (2.94, 3.05)
Basic knowledge of meridians and acupoints	3.02 ± 1.02	3.02 (2.97, 3.08)
Etiology and pathogenesis	2.99 ± 1.02	2.99 (2.94, 3.05)
Four diagnostic methods	3.32 ± 1.08	3.32 (3.25, 3.38)
Differential diagnosis	3.06 ± 1.04	3.06 (3.00, 3.12)
Basic knowledge of prescriptions	2.82 ± 1.05	2.83 (2.77, 2.88)
Preferred disease categories	3.12 ± 1.13	3.12 (3.06, 3.19)
Basic knowledge of traditional Chinese medicine nursing	3.28 ± 0.99	3.28 (3.22, 3.34)
Common traditional Chinese nursing techniques	3.42 ± 1.05	3.43 (3.37, 3.48)
Interpretation of traditional Chinese medical classics	2.73 ± 1.06	2.73 (2.67, 2.79)

### Item-level performance in common TCM nursing techniques

Within the Common TCM Nursing Techniques dimension, item scores varied (Table 3). The highest scores were for moxibustion (3.80 ± 1.10), acupoint plaster application (3.75 ± 1.16), and auricular acupressure (3.74 ± 1.17). The lowest scores were for wax therapy (2.90 ± 1.16), Chinese herbal iontophoresis (2.93 ± 1.17), and acupoint/meridian massage (2.98 ± 1.12).

**Table 3:** Scores for common traditional Chinese medicine nursing techniques (*n* = 1,210).

Item	Overall	95% Confidence Interval
Moxibustion	3.8 ± 1.10	3.80 (3.74, 3.86)
Acupoint injection	3.38 ± 1.25	3.38 (3.31, 3.45)
Gua Sha	3.43 ± 1.12	3.43 (3.37, 3.5)
Cupping	3.47 ± 1.12	3.47 (3.4, 3.53)
Chinese herbal enema	3.65 ± 1.12	3.65 (3.59, 3.72)
Acupoint massage	2.98 ± 1.12	2.98 (2.92, 3.04)
Acupoint Plaster application	3.75 ± 1.16	3.76 (3.69, 3.82)
Auricular Acupuncture	3.74 ± 1.17	3.74 (3.67, 3.81)
Wax Therapy	2.9 ± 1.16	2.90 (2.83, 2.96)
Chinese herbal bath	3.37 ± 1.15	3.37 (3.31, 3.44)
Chinese herbal fumigation	3.51 ± 1.14	3.51 (3.44, 3.57)
Chinese herbal moxibustion	3.5 ± 1.24	3.50 (3.43, 3.57)
Chinese herbal poultice	3.26 ± 1.20	3.26 (3.19, 3.32)
Chinese herbal medicine ointment	3.55 ± 1.14	3.55 (3.49, 3.61)
Chinese herbal iontophoresis	2.93 ± 1.172	2.93 (2.87, 3.00)
Chinese herbal medicine topical application	3.6 ± 1.149	3.60 (3.53, 3.66)

### Item-level performance in advanced TCM clinical practice competency

Scores for Advanced TCM Clinical Practice Competency were lower than those for the techniques dimension (Table 4). TCM nursing rounds had the highest score (3.32 ± 1.11). Items related to evidence-based practice and research showed lower scores, including evidence-based TCM nursing practice (3.00 ± 1.06), standardization research for TCM techniques (2.97 ± 1.07), and new advances in TCM nursing research (2.92 ± 1.06).

**Table 4:** Scores for advanced TCM nursing clinical practice competencies (*n* = 1,210).

Item	Overall	95% confidence interval
TCM nursing rounds	3.32 ± 1.11	3.32 (3.26, 3.38)
TCM chronic disease management	3.10 ± 1.005	3.10 (3.04, 3.16)
Evidence-based practice in traditional Chinese nursing	3.00 ± 1.06	3.01 (2.95, 3.06)
Research on standardization of traditional Chinese medicine techniques	2.97 ± 1.07	2.97 (2.91, 3.03)
Specialized disease-specific nursing based on syndrome differentiation	3.10 ± 1.09	3.10 (3.03, 3.16)
Validation of TCM nursing protocols for key diseases	3.09 ± 1.09	3.09 (3.03, 3.15)
New advances in traditional Chinese medicine nursing research	2.92 ± 1.06	2.93 (2.87, 2.98)

### Training needs for TCM nursing

Regarding preferred training methods (Table 5), case analysis (70.7%) and problem-based learning (PBL) scenario teaching (67.4%) were the most frequently selected options for theoretical training, while on-site demonstration (87.1%) and video-based instruction (76.2%) were the most frequently selected options for skills training. Regarding training content (Table 6), the highest demand was reported for fundamentals of TCM (79.7%) and foundational theory of TCM nursing (79.5%), followed by TCM nursing techniques (72.6%) and TCM wellness/health preservation knowledge (72.1%).

**Table 5:** Demand for traditional Chinese medicine nursing training methods (*n* = 1,210).

Item	Option	Frequency (Percentage)
Theoretical training	Case analysis	856 (70.7%)
	PBL scenario-based teaching	815 (67.4%)
	Micro-lectures	735 (60.7%)
	Flipped classroom	712 (58.8%)
	Group discussions	709 (58.6%)
	Bedside competency training	626 (51.7%)
	Self-study	558 (46.1%)
	Morning handover (5 min)	393 (32.5%)
Skill training	On-site demonstration	1,054 (87.1%)
	Micro-lessons or video demonstration teaching	922 (76.2%)
	Bedside comprehensive competency development	725 (59.9%)
	Role-playing	666 (55.0%)
	Flipped learning	620 (51.2%)
	Operational competition	540 (44.6%)

**Table 6:** Demand for TCM nursing training content (*n* = 1,210).

Item	Number of cases	Percentage
Fundamentals of traditional Chinese medicine	964	79.7%
Fundamental theory of traditional Chinese nursing	962	79.5%
Traditional Chinese medicine nursing techniques	878	72.6%
Traditional Chinese medicine wellness and health preservation knowledge	872	72.1%
Traditional Chinese medicine dietary therapy	826	68.3%
Traditional Chinese medicine nursing routines	734	60.7%
Traditional Chinese medicine nursing condition observation	723	59.8%
Traditional Chinese medicine nursing documentation	699	57.8%
Traditional Chinese medicine disease-specific differentiation and nursing	684	56.5%
TCM nursing rounds	663	54.8%
TCM chronic disease care	656	54.2%
TCM nursing protocols (Key Disease Categories)	622	51.4%
Advances in Traditional Chinese medicine nursing research	616	50.9%

### Factors associated with TCM nursing competency

Univariate analyses showed that hospital type, educational level, number of TCM nursing techniques the nurse could perform, willingness to participate in training, managerial emphasis on TCM nursing training, and perceived training volume/adequacy were significantly associated with overall competency scores (all *p* < 0.05) (Table 7). In the multivariable linear regression model, hospital type (*B* = −0.450, *β* = −0.248, *p* < 0.001), educational level (*B* = 0.353, *β* = 0.196, *p* < 0.001), perceived training volume/adequacy (*B* = 0.192, *β* = 0.205, *p* < 0.001), number of TCM nursing techniques the nurse could perform (*B* = 0.170, *β* = 0.165, *p* < 0.001), willingness to participate in TCM nursing training (*B* = 0.132, *β* = 0.108, *p* < 0.001), and managerial emphasis on TCM nursing training (*B* = 0.201, *β* = 0.175, *p* < 0.001) remained significantly associated with overall competency (Table 8). The model was statistically significant (*F* = 153.931, *p* < 0.001), explaining 47.3% of the variance in TCM nursing competency (*R*^2^ = 0.473; adjusted *R*^2^ = 0.470). The Durbin–Watson statistic was 1.811. No serious multicollinearity was detected, with tolerance values ranging from 0.547 to 0.953 and VIF values ranging from 1.050 to 1.829. Residual diagnostics did not indicate major deviations from normality, linearity, or homoscedasticity.

**Table 7:** Univariate analysis of factors influencing TCM nursing competency.

Item	Option	Number of cases (Percentage)	TCM nursing competency (Score)	Statistical value	*p*
Gender	Male	53	3.26 ± 0.75	0.556	0.578
	Female	1,157	3.19 ± 0.91		
Age	20–30 years old	490	3.25 ± 0.90	1.221	0.301
	31–40 years old	475	3.15 ± 0.90		
	41–50 years old	202	3.15 ± 0.88		
	51 years old and above	42	3.13 ± 1.11		
Years of service	0–5 years	293	3.21 ± 0.81	1.077	0.366
	6–10 years	333	3.30 ± 0.88		
	11–15 years	313	3.18 ± 0.97		
	16–20 years	76	3.00 ± 1.02		
	21 years and above	195	3.05 ± 0.92		
Professional title	Junior	618	3.19 ± 0.92	0.039	0.962
	Intermediate	463	3.19 ± 0.89		
	Associate Professor and above	128	3.21 ± 0.87		
Employment status	Permanent Staff	317	3.20 ± 0.89	0.105	0.916
	Non-staff	892	3.19 ± 0.91		
Education	Technical secondary school	24	1.05 ± 0.06	102.475	<0.001
	Junior College	327	2.96 ± 0.94		
	Bachelor’s degree and above	859	3.33 ± 0.81		
Number of TCM nursing techniques performed	1–2 items	464	2.89 ± 0.91	44.097	<0.001
	Items 3–4	271	3.33 ± 0.88		
	5 or more	474	3.40 ± 0.84		
Training willingness level	Very unwilling	18	3.46 ± 0.98	33.07	<0.001
	Unwilling	30	2.46 ± 0.94		
	Indifferent	83	2.67 ± 0.83		
	Willing	767	3.10 ± 0.78		
	Very Willing	312	3.60 ± 1.03		
Importance of training	Very little emphasis	11	2.72 ± 1.34	97.677	<0.001
	Not important	18	1.80 ± 0.52		
	Average	167	2.36 ± 0.73		
	High	559	3.13 ± 0.71		
	Highly important	455	3.64 ± 0.89		
Number of training sessions	Very insufficient	51	2.34 ± 1.12	125.719	<0.001
	Insufficient	299	2.76 ± 0.8		
	Unclear	156	2.62 ± 0.75		
	Barely sufficient	704	3.56 ± 0.77		

**Table 8:** Multiple linear regression analysis of factors associated with traditional Chinese medicine nursing competency scores.

Variables	*B*	Standard error	Beta	*t*	Significance	95.0% confidence interval for B		Tolerance	VIF
						Lower bound	Upper bound		
(Constant)	0.55	0.20	—	2.70	0.007	0.15	0.95	—	—
1. Type of Hospital	−0.45	0.05	−0.25	−8.75	<0.001	−0.55	−0.35	0.547	1.829
17. Regarding the quantity of traditional Chinese medicine nursing training you currently receive	0.19	0.02	0.21	8.53	<0.001	0.15	0.24	0.953	1.05
7. Education	0.35	0.04	0.20	9.13	<0.001	0.28	0.43	0.76	1.316
16. How would you rate the level of importance your hospital administration places on TCM nursing training?	0.20	0.03	0.18	6.68	<0.001	0.14	0.26	0.599	1.668
10. Number of TCM nursing techniques you can perform	0.17	0.03	0.17	6.09	<0.001	0.12	0.22	0.79	1.266
15. Your level of willingness to participate in TCM nursing training	0.13	0.03	0.11	4.60	<0.001	0.08	0.19	0.64	1.562

The model was statistically significant (*R*^2^ = 0.473, adjusted *R*^2^ = 0.470, *F* = 153.931, *p* < 0.001). The Durbin–Watson statistic was 1.811. No serious multicollinearity was observed (tolerance = 0.547–0.953; VIF = 1.050–1.829).

## Discussion

### Regional status and gaps in TCM nursing competency

In this multicenter survey, overall TCM nursing competency among clinical nurses in Guangyuan City was at a moderate level (mean score 3.19 on a 5-point scale), with clear variation across domains. Competency in common TCM nursing techniques was relatively higher than that in foundational TCM theory and advanced clinical practice, indicating an imbalanced competency profile.

Compared with reports from some tertiary hospitals and TCM hospitals in developed eastern regions, the overall level observed in this study appeared lower, while it was broadly comparable to findings from primary or similar-tier healthcare settings. This pattern may be related to contextual differences in institutional resources, availability of standardized training and practice platforms, and the extent to which TCM nursing services are integrated into routine clinical workflows. In addition, regional variation may also be related to differences in local health policy support for TCM service development, continuing education systems, and academic incentive mechanisms for research engagement, evidence-based practice, and professional advancement. In resource-constrained settings, comparatively limited policy support, fewer structured continuing education opportunities, and weaker academic incentive structures may restrict nurses’ access to sustained competency development. The observed domain imbalance may reflect a more operationally oriented pattern of competency development, whereas theoretical integration and higher-level clinical or academic capabilities appeared relatively less developed and may warrant further attention (37).

### Characteristics of knowledge structure and skill composition

Item-level results further suggested unevenness within the theoretical knowledge domain. Content closely linked to bedside practice (e.g., the four diagnostic methods) was rated higher, whereas items requiring systematic understanding (e.g., interpretation of classical texts and prescriptions/medicinals) were rated lower. One possible explanation is that in-service learning and daily clinical exposure may prioritize practical, problem-oriented knowledge, whereas structured curricula for classical theory and systematic knowledge may be less emphasized or less accessible in routine training.

Within the techniques domain, higher scores clustered in mature, widely implemented techniques with clearer operational procedures and broader clinical applicability (e.g., moxibustion and acupoint plaster application). In contrast, comparatively lower scores for techniques such as wax therapy and herbal iontophoresis may be related to differences in training coverage, fewer opportunities for supervised practice, equipment or safety requirements, and more conservative adoption in certain departments. These findings may suggest that competency differences across techniques are associated not only with training volume but also with variation in technique-specific training pathways and practice opportunities.

Notably, advanced clinical practice competencies—particularly those related to evidence-based practice, standardization research, and keeping up with research advances—showed the lowest ratings overall (19, 38, 39). This pattern may indicate limited exposure to research training, fewer institutional opportunities for evidence-based implementation, and insufficient mentorship or infrastructure to support higher-level competency development beyond routine clinical tasks.

### Organizational and individual correlates of competency

Multivariable analyses indicated that hospital type, educational attainment, perceived training volume/adequacy, managerial emphasis on TCM nursing training, number of techniques the nurse could perform, and willingness to participate in training were independently associated with overall competency (40). These findings suggest that TCM nursing competency may be related to both individual characteristics and the organizational environment.

At the organizational level, different institution types and tiers may vary in training resources, protected learning time, clinical practice platforms, and managerial support, which may in turn be associated with unequal opportunities for skill acquisition and consolidation. At the individual level, higher educational attainment may be related to stronger learning capacity, clinical reasoning, and knowledge integration, while stronger willingness to participate in training may reflect greater engagement with learning opportunities and deliberate practice (41–46). From a nursing management perspective, these findings may be interpreted as suggesting that structural conditions and practice opportunities are linked to competency performance; however, the present cross-sectional design does not allow causal inference.

### Training needs and practical implications

The reported training preferences indicate that nurses favored contextualized and practice-oriented learning formats, particularly case analysis, PBL, and on-site demonstration (47, 48). Meanwhile, the highest demand for training content focused on foundational TCM and TCM nursing theory, followed by TCM nursing techniques and wellness-related knowledge, suggesting perceived gaps in systematic theoretical preparation and standardized technical guidance. Based on these findings, a structured in-service training framework may be considered. Specifically, foundational theory may be strengthened through case-based and PBL-oriented teaching linked to common clinical scenarios; technical competence may be enhanced through on-site demonstration, video-assisted learning, supervised practice, and competency-based assessment; and advanced practice capacity may be supported through evidence-based learning activities, protocol interpretation, and mentorship-supported quality improvement projects (49, 50). At the organizational level, protected training time, managerial support, staged competency evaluation, and inter-institutional collaboration may help improve the feasibility and sustainability of such training efforts. Future longitudinal or interventional studies are needed to determine whether implementation of this framework is associated with measurable improvements in TCM nursing competency.

### Limitations

Several limitations should be noted. First, the cross-sectional design precludes causal inference; longitudinal or interventional studies are needed to clarify temporal relationships and evaluate training effectiveness. Second, participants were recruited from public medical institutions in a single city, which may limit generalizability to other regions or healthcare systems. Third, competency was measured using self-reported instruments, which may be influenced by social desirability and subjective perception; future studies should incorporate objective assessments (e.g., standardized skills evaluation or multi-source appraisal). Finally, residual confounding may remain because several potentially important variables were not measured or controlled for in the present analysis, including training quality, workload, shift patterns, department type, mentorship availability, and departmental culture. Future research should further examine these contextual and organizational factors to better clarify their potential relationships with TCM nursing competency.

## Conclusion

In this multicenter cross-sectional survey of nurses from multi-tier public medical institutions in Guangyuan City, overall TCM nursing competency was at a moderate level with an imbalanced competency profile across domains. Hospital type, educational attainment, number of TCM nursing techniques nurses could perform, and training-related factors (including training adequacy, managerial emphasis on training, and willingness to participate) were independently associated with competency. These findings suggest that TCM nursing competency may be related to both individual characteristics and organizational training conditions. Regionally tailored, competency-based training programs—particularly those targeting foundational theory and advanced clinical practice—together with strengthened institutional support mechanisms, may be considered in future workforce development efforts across different institutional tiers.

## Data Availability

The raw data supporting the conclusions of this article will be made available by the authors, without undue reservation.
